# Effects of Bleaching Gels on Dental Enamel Crystallography

**DOI:** 10.3290/j.ohpd.b875523

**Published:** 2021-01-26

**Authors:** Tatiana Vargas-Koudriavtsev, Pamela Fonseca-Jiménez, Patricia Barrantes-Delgado, Berta Ruiz-Delgado, Geraldine Conejo-Barboza, Óscar-Andrey Herrera-Sancho

**Affiliations:** a Specialist in Dentistry and Prosthodontics, Dental Faculty, University of Costa Rica, San José, Costa Rica; Postgraduate Program in Prosthodontics, University of Costa Rica. Idea, hypothesis, experimental design, wrote the manuscript, proofread the manuscript, performed part of the statistical evaluation, contributed substantially to the discussion.; b General Dentist, Dental Faculty, University of Costa Rica, San José, Costa Rica. Performed part of the experiments, performed part of the statistical evaluation.; c General Dentist, Dental Faculty, University of Costa Rica, San José, Costa Rica. Performed part of the experiments, contributed substantially to the discussion, wrote the manuscript.; d General Dentist, Dental Faculty, University of Costa Rica, San José, Costa Rica. Performed part of the experiments, contributed substantially to the discussion, wrote the manuscript.; e Chemist, Chemistry Faculty, University of Costa Rica, San José, Costa Rica; Centro de Investigación en Ciencia e Ingeniería de Materiales (CICIMA), Universidad de Costa Rica, 11501 2060, San José, Costa Rica. Performed part of the experiments, performed X-ray diffraction (XRD), proofread the manuscript.; f Professor; Physics Faculty, University of Costa Rica, San José, Costa Rica; Centro de Investigación en Ciencia e Ingeniería de Materiales (CICIMA), Universidad de Costa Rica, San José, Costa Rica; Centro de Investigación en Ciencias Atómicas, Nucleares y Moleculares (CICANUM), Universidad de Costa Rica, San José, Costa Rica. Experimental design, performed part of the experiments, interpretation of the results of the XRD readings, proofread the manuscript, contributed substantially to the discussion.

**Keywords:** bleaching agent, enamel crystallography, X-ray diffraction

## Abstract

**Purpose:**

The aim of the present research was to analyse the effects of two bleaching agents, on the enamel crystallography by means of X-ray diffraction.

**Material and Methods:**

Twelve human sound posterior teeth, were collected for the present study (n = 12) and from each tooth two enamel slabs were obtained and randomly assigned to one of two different bleaching protocols. The first protocol involved an in-office bleaching agent (hydrogen peroxide 37.5%/ SDI Polaoffice+), and the second an at-home whitening product (carbamide peroxide 16%/ PHILIPS Zoom! NiteWhite). X-ray diffraction readings were made before and after applying the treatments in order to analyse the peak intensity and crystal domain size. Additionally, scanning electron microscopy (SEM) and energy-dispersive X-ray spectroscopy (EDX) were carried out to identify the composition correctly. Statistical analysis included repeated measures analysis of variance (p ≤ 0.05).

**Results:**

Peak intensity in spectra obtained by X-ray diffraction had a tendency to diminish, mostly in the at-home bleaching group. The analysed data approximate a decrease in the crystal domain size among the samples treated for longer periods of time. Statistical analysis depicted no statistically significant differences among the experimental groups (p ≥ 0.05).

**Conclusions:**

Crystal domain size had a tendency to decrease, mostly when the enamel was treated by bleaching gels that had to be applied by prolonged periods of time.

Teeth whitening may be accomplished by the physical removal of the stain or a chemical reaction to lighten the tooth colour. The active ingredient in most whitening products is hydrogen peroxide (H_2_O_2_),^5^ affecting primarily the dental enamel which is composed mainly of apatites.^14^

From the first description of whitening to date, numerous studies have investigated the effects of hydrogen peroxide and carbamide peroxide on dental tissues. The increase of the roughness after the application of the bleaching agent has been thoroughly investigated by several authors.^4,10,12^ Furthermore, the academic community has extensively explored a decrease in the hardness or microhardness of dental enamel,^12^ which occurs when these treatments are used for prolonged periods.^1^ Prior research generally confirms that the change in the enamel is proportional to the treatment time and the concentration of hydrogen peroxide^3^ and several studies agree that in-office techniques have faster results and shorter application times due to their higher concentrations.^9^

For the analysis of chemical changes caused by bleaching agents on dental surfaces (mainly enamel), different methodologies have been proposed. Using energy-dispersive X-ray spectroscopy (EDX), a reduction in calcium ion levels was observed;^13^ on the other hand, studies in infrared (IR) and Raman spectroscopy, have described phosphate,^3,17^ carbonate^15^ and titanium^16^ alterations after the bleaching treatment. Another method, called X-ray diffraction (XRD), is employed to analyse the crystallographic configuration of materials. However, little research using XRD has been conducted to confirm consistent or conclusive results after the application of hydrogen peroxide or carbamide peroxide.

Nonetheless, previous studies have described the results obtained by XRD qualitatively and not quantitatively.^6,7,11^ Additionally, dental enamel is often studied in its powdered form, which makes it impossible to subject the samples to different treatment variables and using each sample as its own control.

A research group related the crystallite size of dental enamel with the clarity, chroma and hue of the tooth, in other words, they associated the structure of the enamel with its optical properties.^6^ It was found that tooth clarity and hue are inversely correlated with crystal size. Another publication by the same investigators reported that the size of the crystals influences the physical and mechanical macroscopic properties of polycrystalline materials. For instance, the microhardness of the dental enamel has an inverse correlation to the size of the apatite crystals. However, although the hardness is increased by refining the size of the glass, when a critical size is reached below the optimum the mechanical properties decrease dramatically.^7^

The objective of the present study is to analyse the effects of at-home and in-office whitening on the crystal structure of enamel by means of XRD, using a solid and quantitative assessment, by estimating, analysing patterns, peaks, and size of the crystal. The working hypothesis was that the intervention tested would lead to a drop in crystal particle size in both groups.

## Materials and Methods

### Specimen Preparation

The present research protocol was approved by the Research Commission of the Dental Faculty, along with the Vice Rector’s Office for Research at the University of Costa Rica. A sample of 12 human sound posterior teeth, extracted for periodontal or orthodontic reasons, was collected. After cleaning the teeth with a scalpel, these were inspected under magnification for caries and superficial defects and immersed in de-ionised distilled water at 32°C.

Each tooth was sectioned to obtain two enamel slabs by means of a rotating water-cooled saw (Buehler IsoMET 1000 Precision Saw, IL, USA) and a diamond disc (Buehler IsoMET Diamond Wafering Blades, IL, USA). Each one of the two enamel slabs was assigned randomly either to the experimental Group A or B. Also, each slab was stored in a specific container, specifically labelled in order to analyse each specimen separately along the treatment weeks, in accordance to the statistical model explained next.

The specimens were analysed by means of XRD prior to the bleaching procedure, in order to obtain a diffractogram of the sample without treatment. Hence, each enamel slab served as its own control.

### Bleaching Protocol

Table 1 depicts the two experimental groups tested in this study. Group A received an in-office bleaching agent for 8 min, according to the manufacturer’s instructions. Afterwards, the gel was removed with distilled water, dried gently with air and the procedure repeated two more times, for a total of 24 min of exposition. Specimens were stored immediately in distilled water in an incubator at 32°C (VWR Sheldon Mfg 1510E, Sheldon Manufacturing, Cornelius, OR, USA).

**Table 1:** Whitening agents employed in the study

Group	n	Product	Composition	Lot
A	12	SDI Polaoffice+	Hydrogen peroxide 37.5%	1083949
B	12	PHILIPS Zoom! NiteWhite	Carbamide peroxide 16%	16167008

Group B was bleached with a home whitening product for a total of 21 days, according to the manufacturer’s instructions. The product was applied daily for 5 h, after which the peroxide was removed and the specimens were rinsed and stored in distilled water until the next application.

### X-Ray Diffraction (XRD)

Diffractograms were collected for each sample employing a commercially available Bruker model D8 Advance. XRD diffractograms were measured with Cu kα1-kα2 radiation accompanied by a Bragg-Bentano configuration and lineal Lynx-eye detector. Scans in 2Ѳ at a 5–70 scanning angle, 0.019 step size and 384 s scan step time. The database employed to identify and compare the spectra was the PDF-2 from the International Centre for Diffraction Data. For the measurements reported here, samples were not grounded. Before and after bleaching diffractograms were obtained and compared in terms of angle values (x-axis) and intensity (y-axis) for the selected peaks.

In order to estimate crystallite size, as well as lattice strain, X-ray peak profile analysis by modified Williamson-Hall (W-H) models was performed, as described in the literature.^18^ Due to the high data variability, only high intensity diffraction peaks were selected for this analysis. Therefore, the selected diffraction peaks along with the assigned Miller indices were: (002), (102), (211), (212), (213), (202), (004) and (323). The area under the curve for each peak was calculated by means of OriginPro 8.5 software. Standard diffraction files of hydroxyapatite (JCPDS# 09-0432) were used in order to do the indexing of the peaks of each sample.

Assuming that the crystallite strain (ɛ) is isotropic, the total change in the diffraction peaks is given by the formula 1,


$$\beta_{hkl}\cos\theta_{hkl}=\frac{k\lambda }{D_{v}}+4\varepsilon \sin\theta_{hkl}\;(1)$$

where k is a constant shape factor (used usually as k = 0.9) and λ represents the wavelength of the X rays for Cu k_α_ radiation (0.154056 nm), employed by the diffractometer; θ_hkl_ is Bragg diffraction angle (in units of °), D_n_ is the volume weighted crystallite size (in units of nm) and β_hkl_ is the broadening of the hkl diffraction peak measured at half of its maximum intensity (FWHM).

In order to derive microstructural parameters like crystallite size and lattice strain, we explored the linear dependence between the variables by establishing 4sinθ_hkl_ in the x-axis and β_hkl_cosθ_hkl_ in the y-axis.

### Scanning Electron Microscopy and Energy-Dispersive X-ray Analysis

One randomly selected specimen from each experimental group was analysed in a scanning electron microscope, SEM (model S-3700N, HITACHI). Samples were coated with gold with a thickness of 25 nm. The readings were carried out at 15kV accelerating voltage with a working distance of 10 ± 0.1 mm. The acquisition mode used while carrying on EDX measurements was ‘Auto’, which enables acquisition to continue until enough counts are collected in the spectrum for quantification. This mode also helps to reach the accuracy reported by the manufacturer which is better than 0.1wt% and in order to systematise the acquisition process for each sample. The area tested was of approximately 240 µm^2^.

### Statistical Analysis

Three selection criteria were employed to choose the specimens for the statistical analysis: the first criteria stated that only specimens that were considered to have a peak pattern similar to hydroxyapatite/fluorapatite^18^ would be analysed. Second, only peaks with an intensity higher than 400 arbitrary units would be considered. The third requisite was that the selected peaks had to be present in the spectra before and after bleaching in at least eight specimens in each experimental group.

The calculated difference in angle and intensity values was analysed by means of descriptive statistics (mean value, standard deviation and confidence intervals). Angle values for each peak before and after whitening were subtracted. A positive difference means that the angle decreased after bleaching (shift to the left), whereas a negative difference means an increase in the angle value after treatment. The same was calculated for the peak intensity values.

One-way analysis of variance was carried out (p ≤ 0.05) to determine whether the differences among the FWHM of the peaks at 32°, 49°, 53° and 64.3° angles in the two experimental groups were statistically significant.

## Results

**Fig 1:**
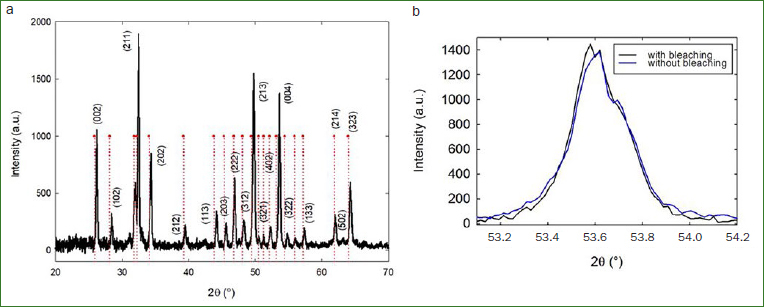
(a) Spectra obtained by XRD, before the whitening procedure. (b) presents the peak correspondent to the Miller index 004 before (blue) and after (black) bleaching. The dotted lines correspond to hydroxyapatite (JCPDS# 09-0432).

Figure 1 corresponds to one of the typical XRD spectra of the samples before being treated with the bleaching agents. The x-axis corresponds to the reflected beam at an angle 2-theta (2q), where the diffraction signal was detected. On the y-axis is intensity with which that signal was detected.

A well-defined peak pattern can be seen, due to the atomic periodicity of the analysed structure. Every peak is marked with the corresponding Miller index. These are used to describe planes and directions within the crystal structure. The Miller indices for a plane are represented by the nomenclature (h, k, l) where the parentheses indicate that it is a plane and the values of h, k, and l relate the plane intercepts to the axes system.

Using the XRD database PDF-2 of 2007 ICDD International Centre for Diffraction Data, the pattern was equivalent to hydroxyapatite and/or fluorapatite. The latter was due to the close proximity between the diffracted angles values from the two minerals.

Scanning electron microscopy and X-ray energy dispersion were used in one sample from each experimental group to perform a morphological characterisation and an elemental analysis.

**Fig 2:**
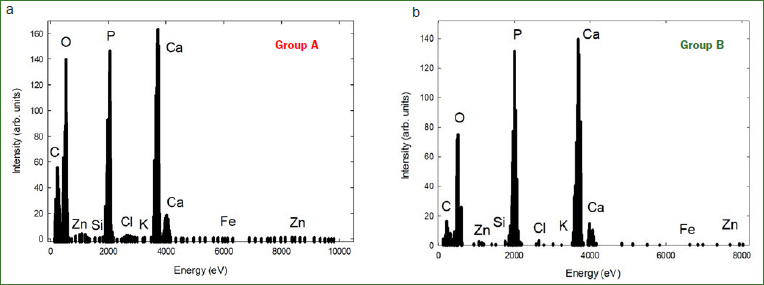
General EDX analysis of the chemical elements found and its abundance in both experimental groups.

The left panel of Figure 2 depicts a histogram with the elements found and its respective abundance in Group A and the right panel corresponds to Group B. As can be seen from the histogram, calcium and phosphorus were the most plentiful. A prevalence of calcium, phosphorus, oxygen and carbon was detected.

It is important to note the correlation that exists between the highlighted points in Ca and P. This relation makes it possible to determine the ratio that exists between them, which is approximately 1.7. The ratio Ca/P in hydroxyapatite obtained in our measurements agrees with other authors within less than 1%.^8^ The results obtained using EDX do not show any peak at 0.677 keV where we expected to have the characteristic X-ray emission K_a_ line for fluorine. As can be seen from the data in Figure 2, no counts were detected in this range. Consequently, the findings of this study suggest that the crystal correlates to hydroxyapatite.

**Fig 3:**
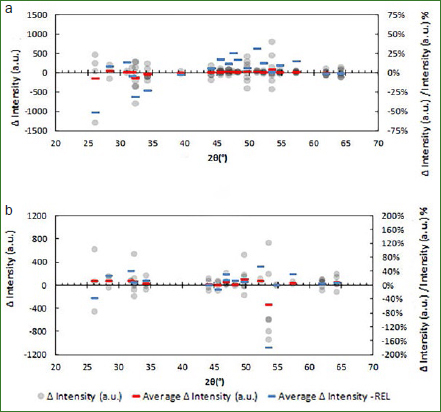
Statistical analysis of the intensity of the peaks.

Figure 3 depicts the spectrum analysis relating peak intensity behaviour for experimental Group B before and after bleaching.

For visualisation purposes these differences are grouped according to the mean angle position of the peaks (x-axis), where every grey dot represents the subtraction between the intensities before and after the treatment. The red lines represent the mean of these differences and the blue line corresponds to the mean relative difference based on the initial unbleached condition.

It can be seen a general tendency of the means to be positive, which indicates that the peak intensity mostly diminishes. Although most results follow the same scheme, the only statistically significant change in peak intensity (using two tails t-confidence intervals with a threshold of 5%) was found on the peak in the 48.3° angle. This diminishing tendency was also found in the Group A, but to a lesser degree.

Calculations are given for 14 peaks, however, analysis of variance for comparison among the two experimental groups is made with only four peaks for the reasons mentioned in the former section. Also, some peaks, like the (212) peak, were excluded because there were samples where the amplitude was very small to measure realistic amplitudes.

The same analysis was performed with the angle values in order to determine if there was a tendency to increase or decrease, but the results showed that changes were smaller than the resolution of the method used to determine the peak angle; furthermore the dispersion of the data made it difficult to determine any kind of tendency.

After the statistical analysis, mechanical properties like particle size, lattice strain and deformation stress were obtained using the Miller indices and the corresponding broadening of the hkl diffraction peak measured at full-width at half-maximum (FWHM). To obtain these properties the following graphs were generated, using 4sinθ_hkl_ along the x-axis and β_hkl_cosθ_hk_ along the y-axis.

**Fig 4:**
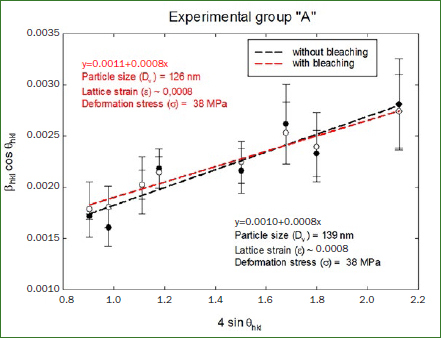
Williamson-Hall profiles of Group A.

In Figure 4, the office bleaching treated samples (Group A) showed a drop of 9% in crystal particle size from 139 nm to 126 nm, while the deformation stress remained the same at 38 MPa.

**Fig 5:**
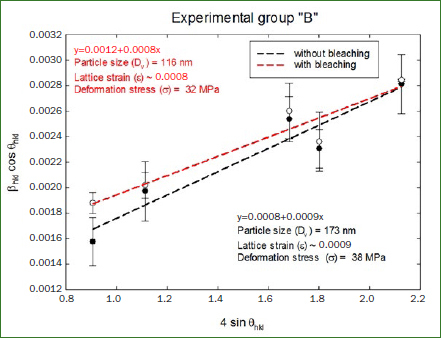
Williamson-Hall profiles of Group B.

In Figure 5, the house bleaching samples showed a greater change (33%) in the particle size going from 173 nm to 116 nm. Contrary to the office bleaching, the deformation stress changed from 38 MPa to 32 MPa (about 16% less).

In the linear fit, the coefficients of determination (R^2^) for Group A were 0.9212 and 0.9687 before and after whitening, and for Group B 0.9558 and 0.9534.

**Fig 6:**
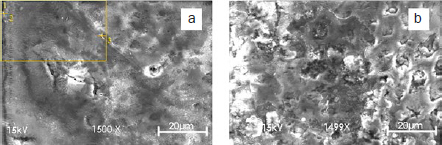
Scanning electronic microscopy for dental enamel after applying office and house whitening.

These results can be also illustrated, with SEM images in Figure 6. In both images the external surface of the dental enamel is observed. Panel (a) with the smoother surface corresponds to office whitening, while panel (b) with a rougher surface corresponds to that of the house whitening treatment.

In Table 2, none of the p-values is smaller than the threshold 0.05. This implies that when comparing the FWHM in 32°, 49°, 53° and 64.3° angles in the samples bleached with house treatment and those bleached with office treatment no statistically significant variation was found that implies a difference in the mechanical properties of the enamel between them.

**Table 2:** ANOVA analysis for Miller indices 211, 213, 004 and 323

Angle	Miller indices	Statistical significance*
32°	(211)	0.83
49°	(213)	0.19
53°	(004)	0.99
64.3°	(323)	0.79

*p ≤0.05.

## Discussion

The specimens in our sample were characterised as hydroxyapatite combining the information obtained by means of XRD and EDX. Although the pattern produced by the XRD could be either characterised as fluorapatite or hydroxyapatite, as stated by other authors,^20^ the results of only these measurements do not explain which is the predominant crystal in the samples. Hence EDX measurements were carried out on only two specimens in order to elucidate the crystalline structure of our samples. As can be seen from the EDX data, no counts were detected in the range of 0.677 keV where we expected to have the characteristic X-ray emission K_a_ line for fluorine. Consequently, these findings and the ratio between Ca and P finally identified the crystal as hydroxyapatite.

To the authors’ knowledge, the present *in-vitro* research is one of the first attempts to study in a quantitative manner the effects of tooth bleaching on dental enamel by means of XRD. The enamel slabs analysed in this investigation were not ground into powder, but rather kept in its intact form in order to perform the diffraction readings before and after bleaching, so that each sample becomes its own control. Prior studies have failed to evaluate/identify the same specimen before and after applying a surface modification agent. The latter due to the fact that previous research has focused on enamel in its powdered form.

It could be observed that after applying the whitening agent according to the manufacturer’s instructions, both experimental groups depicted a diminished crystal size. This goes in accordance to another study,^6^ which showed that the size of enamel crystals was inversely proportional to tooth lightness. The authors explain that the smaller enamel crystals increase the amount of light scattered from the surface and thus the substratum appears lighter.

Also, in the present study it is worth noting that the at-home bleaching agent (Group B) which had to be applied for more sessions and a longer period of time, caused a greater reduction in the crystal size, in comparison to the in-office bleaching (Group A), which was applied for three sessions in accordance with the manufacturer’s instructions. This might suggest that the enamel structure and composition is rather affected by the prolonged application time than by the concentration of the bleaching agent. However, it would be advisable to compare application times using the same bleaching agent at the same concentration to be able to conclude this in a more definitive manner.

Previous investigations by our group showed that at-home bleaching agents applied for longer periods of time decrease the amount of carbonate and titanium molecules in dental enamel.^15,16^ This goes in concordance with the results published by Eimar et al^6^ that proved that the carbonate content is inversely correlated with tooth lightness, and therefore with the crystal size.

Another research published by Eimar et al^7^ stated that the enamel crystal size was also related with its mechanical properties. The former authors concluded that a smaller crystal size correlates with a greater microhardness, however, if a critical size is reached, the mechanical properties will be affected dramatically. Although our investigation failed to prove a statistically significant difference in the deformation stress before and after bleaching, the at-home whitening gel decreased this characteristic in 16%. This could also be observed clinically, because all specimens in Group B appeared to be brittle after the treatment.

It is worth mentioning other investigations that describe the possible correlation between the lightness of hydroxyapatite and its grain size. Wang and Shaw^19^ discuss in their article that some forms of hydroxyapatite might have smaller grain sizes (mainly distributed between 50 and 110 nm) and that there might be nanopores in between the crystals. This pores scatter light effectively since their refractive index is approximately 1, in comparison to hydroxyapatite (1:65). Hence, if a whitened tooth decreases its crystallite size, maintains its volume, and becomes more porous, this contributes to its lighter appearance. Apetz and van Bruggen^2^ also analyse in their research the role of grain boundaries in light scattering. They discuss that materials with smaller crystals present more grain boundaries, which also have the property of increasing light scattering. This illustrates the complexity of the tooth whitening process, which apparently is not only related to changes at the molecular level, but also to the crystallite size of hydroxyapatite and the relationship between its grains and resultant nanopores.

A drawback of our framework is the high variability in the measured data. This occurs because the extracted samples belong to different individuals and thus present different crystallographic features. Although there was a clear tendency to decrease the particle size and deformation stress, mostly in Group B, the high variability precluded a statistically significant result. This goes in accordance with other observations in the published literature.^6,7^

## Conclusions

The findings of this research suggest a decrease in the enamel crystal size after the application of bleaching agents. This reduction is more notorious when a whitening product was applied for a longer period of time (at-home bleaching), than when it was applied for fewer sessions (in-office bleaching). This study also depicted a high variability regarding the crystallographic properties among the samples.

### Conflicts of Interest

There are no potential conflicts of interest with respect to the authorship of this article.
